# Early Diagnosis of Pathogen Infection by Cell-Based Activation Immunoassay

**DOI:** 10.3390/cells8090952

**Published:** 2019-08-22

**Authors:** Erez Bar-Haim, Shahar Rotem, Uri Elia, Adi Bercovich-Kinori, Ma’ayan Israeli, Inbar Cohen-Gihon, Ofir Israeli, Noam Erez, Hagit Achdout, Ayelet Zauberman, Moshe Aftalion, Emanuelle Mamroud, Theodor Chitlaru, Ofer Cohen

**Affiliations:** 1 Department of Biochemistry and Molecular Genetics, Israel Institute for Biological Research, 74100 Ness-Ziona, Israel; 2 Department of Infectious diseases, Israel Institute for Biological Research, 74100 Ness-Ziona, Israel

**Keywords:** Early detection, *Francisella tularensis*, *Bacillus anthracis*, *Yersinia pestis*, Influenza virus, ELISPOT, lymphocyte activation, infection biomarkers, lymphocyte transcriptomics, animal models

## Abstract

Diagnostic identification of pathogens is usually accomplished by isolation of the pathogen or its substances, and should correlate with the time and site of infection. Alternatively, immunoassays such as enzyme-linked immunosorbent assay (ELISA) tests for quantification of serum antibodies are expedient and are usually employed for retrospective diagnostic of a particular infective agent. Here, the potential of cell-based immunoassays for early pathogen detection was evaluated by quantification of specific, antigen-activated, low-frequency IFNγ-secreting cells in mouse spleens following infection with various pathogens. Using enzyme-linked immunospot (ELISPOT) assays, specific responses were observed within 3–6 days following infection with *F. tularensis*, *B. anthracis*, *Y. pestis*, or Influenza virus. Blood samples collected from *F. tularensis*-infected mice revealed the presence of IFNγ-producing activated cells within one week post infection. When non-human primates were infected with *B. anthracis*, cellular response was observed in peripheral blood samples as early as five days post infection, 3–5 days earlier than serum antibodies. Finally, the expression pattern of genes in splenocytes of *F. tularensis*-infected mice was inspected by a transcriptomic approach, enabling the identification of potential host targets for the future development of genetic-based cellular immunoassays. Altogether, the data demonstrate the potential of cell-based immunoassays for early pathogen detection.

## 1. Introduction

Infectious diseases are specifically diagnosed by direct sampling of the pathogen as a whole, its genetic materials or shed/secreted antigens. Alternatively, various immunoassays can detect the immune response elicited by a pathogen late in infection, providing a retrospective confirmation of the exposure to that pathogen (e.g., seroconversion). Direct sampling and pathogen identification present several disadvantages. The presence of the pathogen or its substances is limited due to pathogen clearance. In addition, the pathogen or its subunits may be present in the sampled host tissue, but at sub-detectable levels.

Immunoassays are designed to detect the pathogen-specific immune response elicited in the host. While the innate response is activated immediately following incidence of infection, it is not pathogen-specific. The specific acquired immunity is manifested at a later stage following exposure, usually requiring more than one week to reach detectable levels. The acquired response typically includes the humoral immune response, consisting of antibody secretion, and the cellular response that includes cytotoxic activity of CD8+ T cells and cytokine secretion by both CD4+ and CD8+ T cells. The acquired response can last for years following priming [1]. Nascent humoral response is characterized by the IgM antibody isotype, later converting mainly to IgG and IgA. In the case of T cells, the response may be characterized by the modulation of several surface markers. Naïve cells are characterized by the expression of both CD27 and CD45RA, while CD27 is expressed by memory cells and CD45RA is expressed by effector T cells [2,3]. CD45RA- memory cells are further divided in two functional subsets, central memory cells express CD62L and CCR7 to allow lymph node homing, while effector memory lack the expression of both [4]. Long-lived, antigen-specific, and naïve-like T cells were also described, designated stem cells memory and characterized by the expression of stem cell antigen 1 (SCA1), the memory markers interleukin-2 receptor β (IL-2Rβ), and chemokine C-X-C motif receptor 3 (CXCR3) [5].

The most common method for quantification of an antibody immune response is enzyme-linked immunosorbent assay (ELISA). In its simplest setup, antibody titers are determined by direct binding to antigen-coated plates. Specific recognition of antigen is visualized by a secondary antibody that is typically conjugated to chromogenic enzymes such as horseradish peroxidase (HRP) or alkaline phosphatase (AP). A simpler and highly cost-effective method is represented by lateral flow assays which can provide qualitative information, usually with a limited sensitivity of detection. While ELISAs aim to measure total analyte concentrations, the number of antibody-secreting cells (ASC) can be evaluated by ASC- enzyme-linked immunospot (ELISPOT) assays. In these assays cells are plated on antigen-coated membranes and spots of bound antibodies are detected by a chromogenic secondary antibody.

The detection and quantification of cellular immune response is more complicated than that of antibody level and is carried-out by various approaches which interrogate the antigen-dependent activation-state of cells. Lymphocytes activation is manifested by various cellular processes, such as cell proliferation, changes in cell shapes, gene expression and protein secretion [6], and therefore may be evaluated by various methods. Secreted cytokines may be quantified individually by methods such as ELISA or in multiplex assays, to provide information on several analytes in a single, low volume assay. Cytokine-producing cells may be enumerated by methods such as ELISPOT and flow cytometry following intracellular staining. While ELISPOT is simpler to perform and does not require sophisticated instruments, intracellular staining is more informative, facilitates the detection of several cytokines in one staining and may provide additional information such as cell type, activation and memory status, etc.

ELISPOT allows the recognition of low-frequency responding cells (usually at least 1:100,000) by detection of secreted proteins, such as cytokines and growth factors, at the single-cell level. The assay may be performed with frozen peripheral blood mononuclear cells (PBMC), enabling flexibility of sample processing [7]. The assay was developed originally as a method to detect specific antibody-secreting cells, and later adopted to characterize other lymphocyte responses [7,8]. It is extensively used for immune response characterization in various areas of medical research, including basic immunological studies, vaccine development, infectious disease, autoimmunity and allergy diagnostic. Although most immunoassays for pathogen detection in clinical samples are aimed at the detection of antibody response [8], there are a few examples for the clinical use of ELISPOT for immunodiagnosis, the most familiar one being the diagnosis of tuberculosis [9]. Tuberculin skin test was replaced by IFN-γ release assay (IGRA), which scores secretion of the cytokine ex vivo in response to tuberculosis antigens. The use of stimulating protein antigens of *Mycobacterium tuberculosis*, that are not expressed by the vaccine strain *Bacillus Calmette-Guérin* (BCG), allows the identification of a disease-specific immune response [9]. The commercially available “T-Spot.TB” kit is the first example of an ELISPOT kit for the identification of an infectious disease. The ELISPOT assay is currently applied for the detection of additional pathogens, among them viruses, bacteria, protozoa and helminths. To the best of our knowledge, commercial ELISPOT-based kits are available for tuberculosis (TB) [10] and cytomegalovirus (CMV) [11] detection.

Our current study is aimed at exploring the potential of a cell-based immunoassay to serve for diagnosis of pathogen infection, with emphasis on emerging diseases and biothreat agents. We employed the IFNγ secretion ELISPOT assay to characterize the early immune response developed in the spleen subsequent to murine infection with *Francisella tularensis*, *Bacillus anthracis*, *Yersinia pestis* and Influenza. Various types of antigens for lymphocytes stimulation, including fully intact inactivated *F. tularensis* and *Y. pestis*, *B. anthracis* core antigens and the PA protein, and MHC-binding peptide epitopes of Influenza, were used. Specific ELISPOT responses were observed 3–4 days following *F. tularensis* infection, 4 days following *Y. pestis* and *B. anthracis* infection, and 6 days following Influenza infection. Circulatory lymphocyte response could be detected in blood samples collected 7 days following *F. tularensis* infection. In order to examine the applicability of the assay to human samples, peripheral blood mononuclear cells (PBMC) from non-human primates (NHP) were analyzed, revealing specific ELISPOT response as early as 5 days post infection with *B. anthracis*, at least 3 days earlier than the detection of antibody response to the PA and core antigens. Finally, potential markers for future gene-expression-based assays for early identification of pathogen infection, were identified by determining the gene expression signature of immune-primed, antigen-stimulated lymphocytes by a global RNA-seq transcriptome analysis.

## 2. Materials and Methods

### 2.1. Bacteria and Viruses

#### 2.1.1. Francisella Tularensis

Subsp. *holarctica* strain LVS (ATCC 29684) and *F. tularensis* subsp. *tularensis* strain SchuS4 were used for infection. Bacterial glycerol stocks that had been stored at −80 °C were streaked onto cysteine heart agar (CHA) (Becton Dickinson, France) and incubated for 1–2 days at 37 °C. Bacterial cultures were grown at 37 °C in to mid-log phase (optical density of 0.1–0.2 at 660 nm) in TSBC (TSB Difco, supplemented with 0.1% cysteine, BD, France) for LVS and PPB (Bacto proteose peptone, Difco; supplemented with 1% Glucose, 0.5% NaCl and 0.05% cysteine, Sigma-Aldrich, Israel) for SchuS4 strain. The bacteria were washed and re-suspended in PBS, and mice were infected with 10^2^ or 10^5^ bacteria in a volume of 25 µL via the intranasal route after anesthesia with ketamine and xylazine.

#### 2.1.2. Bacillus Anthracis

A Sterne-based sub-strain mutated in the genes for *htrA*, *lef* and *cya* (Sterne *ΔhtrA lef^MUT^ Δcya*) [12] was used in this study. Bacteria were grown and prepared as described before [12,13]. Prior to infection, spore preparations were heat-shocked (70 °C, 20 min) to kill the residual vegetative bacteria. Mice were infected subcutaneously with 10^6^ spores in 0.1 mL of saline, and monkeys were infected subcutaneously with 10^9^ spores in 1 mL saline, as described [12,13].

#### 2.1.3. Yersinia Pestis

Subsp. EV76 bacteria were grown on brain heart infusion agar (BHIA, Difco) for 48 h at 28 °C, suspended in 5 mL saline solution (0.9% NaCl), and then diluted in saline solution to 10^8^cfu/mL. Mice were infected in the subcutaneous route with 10^7^ cfu.

#### 2.1.4. Influenza

Strain A/Puerto Rico/8/1934(H1N1) Influenza virus (PR/8) was a kind gift from Dr. Michal Mandelboim (Central Virology Laboratory, Ministry of Health, Chaim Sheba Medical Center, Tel-Hashomer, Israel). The virus was propagated in embryonated chicken eggs as described previously [14] and virus titer was determined by plaque assay on MDCK cell monolayers. Mice were anesthetized as described above and infected by intranasal instillation with 20 pfu in a volume of 50 µL.

### 2.2. Animals

All animal experiments were approved by the IIBR committee for animal research. The experimental animals were handled according to the National Research Council 1996 Guide for the care and Use of Laboratory Animals and regulations of the IIBR Animal Use Committee. The IIBR animal-experiment protocol were M-64-13 (December 2013), M-40-13 (September 2013), M-36-16 (May 2016), M-73-15 (November 2015), M-07-16 (January 2016) and RM-08-14 (September 2014).

Female C57BL6 mice (6–8 weeks old) were obtained from Jackson. The mice were allowed free access to water and rodent diet (Envigo, Israel).

Female non-human primates (NHP, *Macaca mulatta*, 4.5–5.5 kg) were obtained from RC Hartelust BV, the Netherlands. NHP were quarantined upon arrival and screened for evidence of disease. The animals were individually housed in stainless-steel cages in animal rooms that were maintained at 20–22 °C and relative humidity of 50 ± 10% on a 12-h light-dark cycle.

### 2.3. ELISPOT Assays

Spleens were dissociated in GentleMACS C-tubes (Miltenyi Biotec), filtered and separated on lympholyte-M media according to the manufacture protocol. Mouse PBMC were diluted 1:2 in RPMI media prior to separation on lympholyte-M media. NHP PBMC were separated in Vacutainer^®^ CPT™ Mononuclear Cell Preparation Tube and serum was collected in BD Vacutainer^®^ Rapid Serum Tube (BD Biosciences, Franklin Lakes, NJ, USA). Single-cell suspensions were seeded into 96-well ELISPOT plates (MAIPS4510, Merck Millipore, Ireland) in complete RPMI medium, supplemented with 10% heat-inactivated fetal calf serum, 1 mM Pen-Strep, nonessential amino acids, 2 mM l-glutamine, 1 mM sodium pyruvate, 25 mM HEPES and 5 × 10^−5^ M β-mercaptoethanol. All tissue culture solutions were obtained from Biological Industries (Bet Haemek, Israel). Cells were plated in concentration of 10^6^/well and serially diluted to enable single spot enumeration. Each sample was tested in duplicate. Unless stated otherwise, animals were examined individually in groups of 3.

The following antigens were used for stimulation: UV-inactivated LVS (5 × 10^7^ cfu equivalents/mL); *B. anthracis* urea-extracted protein preparation as previously described [12] (core antigen, 1 µg/mL); *B. anthracis* recombinant PA protein (LPS free, 1 µg/mL); formalin-inactivated *Y. pestis* EV76 (5 × 10^6^ cfu equivalents/mL); Influenza PR8-derived peptides:

1) NP_366–374_ ASNENMETM (D^b^-restricted)
2) PA_224–233_ SSLENFRAYV (D^b^-restricted)
3) NP_311–325_ QVYSLIRPNENPAHK (I-A^b^-restricted)
4) NA_161–175_ SVAWSASACHDGMGW (I-A^b^-restricted)

For all the assays antigen-free cells were used as a negative control, supplemented with medium.

The frequency of IFNγ-secreting cells was determined using ELISPOT kits (Mouse IFNγ ELISPOT Ready-SET-Go!, eBioscience, San Diego, CA, USA) with strict adherence to the manufacturer’s instructions.

### 2.4. ELISA

The ELISAs for the quantification of anti-PA antibodies in the serum of infected NHP were performed as previously described [12]. The ELISA for the quantification of anti-bacterial antibody titers in the circulation of vaccinated animals were performed on microtiter plates coated with a *B. anthracis* urea-extracted protein preparation (core antigens) or recombinant PA as previously described [12].

### 2.5. RNA-Seq

Total RNA was extracted from splenocytes using RNeasy Mini Kit (QIAGEN, Hilden, Germany) according to the manufacturer’s protocol. The extracted RNA was quantified and the RNA quality was assessed using Bioanalyzer with the RNA high sensitivity kit (Agilent, Santa Clara, CA, USA). RNA integrity number (RIN) was calculated and samples with RIN value > 8.0 were sent to Columbia Genome Center (NY, USA) to conduct the RNA-seq process. Libraries were constructed using the TruSeq RNA library preparation kit (Illumina, San Diego, CA, USA) and a whole transcriptome sequencing (total RNA-seq) using an Illumina HiSeq was performed. For each sample over 30 million single 100 nt reads were generated. The expression patterns were examined for genes that were significantly changed (calculated *p* value < 0.05) that showed a change greater than 2 fold. Go enrichment analysis was done using GeneAnalytics database (geneanalytics.genecards.org) [15]. The transcriptomics data are available on line as SRA submission PRJNA544177.

### 2.6. Real-Time PCR

Total RNA was extracted using RNeasy Mini Kit (QIAGEN) according to the manufacturer’s protocol. cDNA was prepared using High-Capacity cDNA Reverse Transcription kit (ABI, Waltham, MA, USA) according to the manufacture protocol. Real time PCR was performed using the SYBR Green master-kit (ABI) with the following primers (forward, reverse):

*IFNγ*: TCAAGTGGCATAGATGTGGAAGAA, TGGCTCTGCAGGATTTTCATG
*Mmp9*: GCCGACTTTTGTGGTCTTCC, AGCGGTACAAGTATGCCTCTG
*Hprt*: TGAAGAGCTACTGTAATGATCAGTCAA, AGCAAGCTTGCAACCTTAACCA

### 2.7. Statistical Analysis

Student’s t test was used for statistical analysis. A *p* value of ≤ 0.05 was considered statistically significant.

## 3. Results

### 3.1. Characterization of Early Immune Response Following F. Tularensis Infection

*F. tularensis* (FT) is an intracellular pathogen that elicits both cellular and humoral immunity [16]. The live vaccine strain (LVS) and Schu strains serve as a disease model in mice, with the latter being more virulent. In a previous work [17] we observed early T cell activation following intranasal FT LVS infection in mice. Activation of T cells, as evidenced by CD69 up-regulation was detected in lungs and mediastinal lymph nodes on day 2 post lethal infection and on day 6 following sub-lethal infection. Additionally, IFNγ gene expression was observed 4 days post sub-lethal infection in pulmonary T cells. The goal of the current study was to probe the validity of cellular immune responses as an early indication for infection.

**Figure 1:**
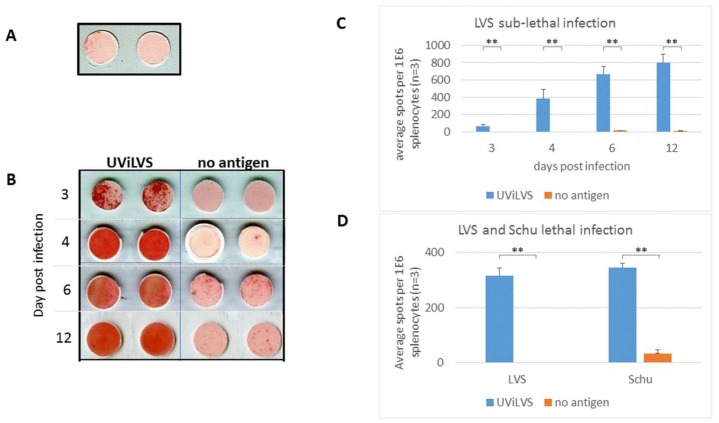
IFNγ ELISPOT response in the spleen following intranasal infection with *F. tularensis*. Mice were infected with a sub-lethal dose (10^2^ cfu, 0.1 LD_50_) of LVS strain, or with a lethal dose of 10^5^ cfu LVS or 10^2^ Schu strain (100 LD_50_). Animals were sacrificed and splenocytes were stimulated in situ in an ELISPOT assay with UV-inactivated LVS (UViLVS, 5E7 cfu equivalent/mL) or left untreated (no antigen). Images of 10^6^ cells/well of splenocytes from naïve (**A**) or sub-lethal LVS-infected mice (day 3 post infection) (**B**) are presented. (**C**) Quantification of IFNγ spots represent groups of 3 mice, collected in the indicated time-points following sub-lethal LVS infection, and individually assayed as described above. (**D**) Three mice were collected 3 days following lethal intranasal infection with LVS (10^5^ cfu) or Schu strain (10^2^ cfu), and assayed as described above. Data are representative of three (LVS infections) and two (SchuS4 infections) independently performed experiments. ** *p* < 0.01.

The systemic immune response in the spleen following sub-lethal respiratory FT infection was characterized in mice infected intranasally with 100 cfu (0.1 LD_50_) of FT of the LVS strain. Groups of 3 mice were inspected 3, 4, 6 or 12 days post infection, allowing the observation of the early disease phase, its peak and the clearance phase. Splenocytes were stimulated in the presence of UV-inactivated FT LVS (UViLVS) bacteria in an ELISPOT assay to detect IFNγ-secreting cells. More than 10 naive mice were examined in the course of the study and their response never exceeded 5 spots per 10^6^ splenocytes (Figure 1A). In infected mice, approximately 70 and 400 spots representing individual IFNγ-secreting cells per 10^6^ splenocytes, were detected on days 3 and 4 post infection, respectively (Figure 1B,C). Control groups (no antigen stimulation) exhibited less than 5 spots, reflecting spontaneous IFNγ secretion. On days 6 and 12 post infection, a limited background response of 10–20 spots without stimulating antigen was observed, significantly lower than the response of antigen-stimulated splenocytes (Figure 1B,C). Altogether, a substantial ELISPOT signal was observed following infection, with limited background response.

Examination of the response following lethal FT intranasal infection (LVS strain, 10^5^ cfu, 100 LD_50_; Figure 1D) established that three days following LVS infection there were more than 300 IFNγ spots per 10^6^ splenocytes. Following lethal infection with the highly virulent SchuS4 strain (10^2^ cfu, 100 LD_50_; Figure 1D), approximately 350 spots were detected in the group of antigen-stimulated splenocytes, compared to 10–20 spots of background response without antigen stimulation.

It may be concluded that in all the examined mice, the antigen-specific response was significantly stronger than that of non-specific response or the response to the stimulating antigen in non-infected mice. Therefore, ELISPOT can serve as a useful tool for monitoring splenocyte response following both sub-lethal and lethal infections, starting at day 3 post infection with FT.

### 3.2. Early Response Following Bacillus Anthracis and Yersinia Pestis

The immune response in the spleens was then addressed following infection of mice with two other pathogens, *B. anthracis* (BA) and *Yersinia pestis* (YP).

**Figure 2:**
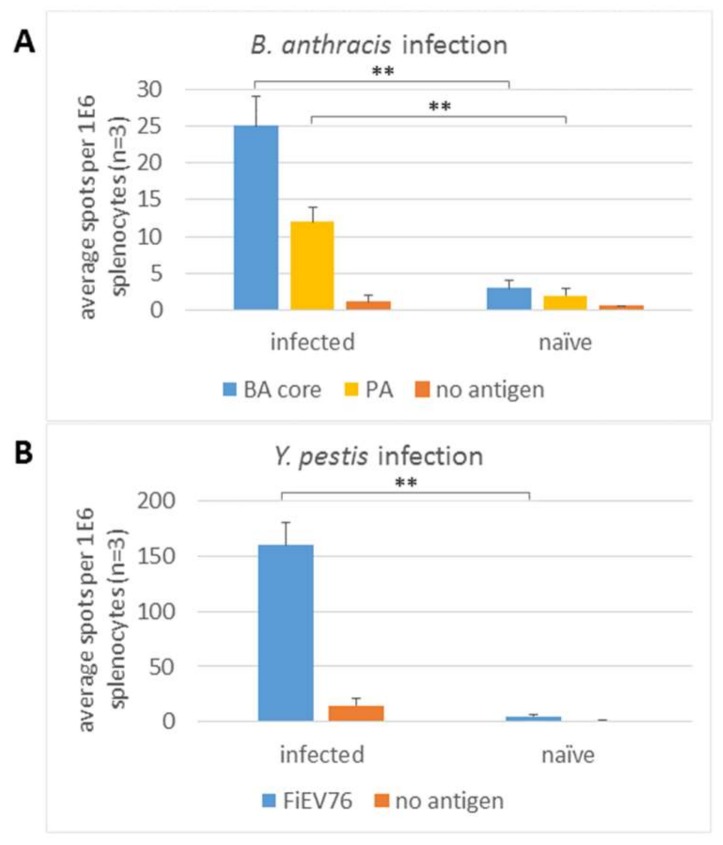
IFNγ ELISPOT response in the spleen following subcutaneous infection with *B. anthracis* and *Y. pestis*. Mice were infected with *B. anthracis* strain Sterne *ΔhtrA lefMUT Δcya* (**A**) or *Y. pestis* subsp EV76 (**B**). Four days later splenocytes were stimulated with *B. anthracis* core antigens and PA protein or formalin-inactivated EV76, respectively. Data are representative of three independently performed experiments. ** *p* < 0.01.

Four days post infection with the Sterne *ΔhtrAlef^MUT^Δcya* live attenuated vaccine strain [12,13] approximately 25 IFNγ spots per 10^6^ splenocytes were observed upon stimulation of splenocytes with a mixture of *B. anthracis* antigens (urea-extracted *B. anthracis* antigens, to be referred as “core” antigen, [12,18]) and 10-15 spots when stimulated with the PA protein (Figure 2A). For both core and PA antigens, no response was observed in naïve, non-infected mice. The early response to the highly immunogenic PA antigen demonstrates the potential of such immunogenic protein antigens for the early detection of infection. It should be noted that the core antigen was prepared from bacteria that do not express PA protein, therefore the core-specific response is independent of that elicited by PA and may be implemented in the case of infection with PA-defective or modified strains.

Splenocytes from YP EV76 infected mice were stimulated four days post infection with formalin-inactivated YP (FiEV76) bacteria (Figure 2B). Upon FiEV76 activation, splenocytes collected from YP-infected mice exhibited 10-times more IFNγ-producing cells compared to the background response of naïve mice. For both bacterial infections (BA and YP), the response of splenocytes from infected mice that were stimulated without antigen was below 5 spots per 10^6^ splenocytes. It can be concluded that the splenocyte response is observed as early as four days post infection with two additional pathogenic bacteria.

### 3.3. Early Response to Peptide Antigens Following Influenza Infection

**Figure 3:**
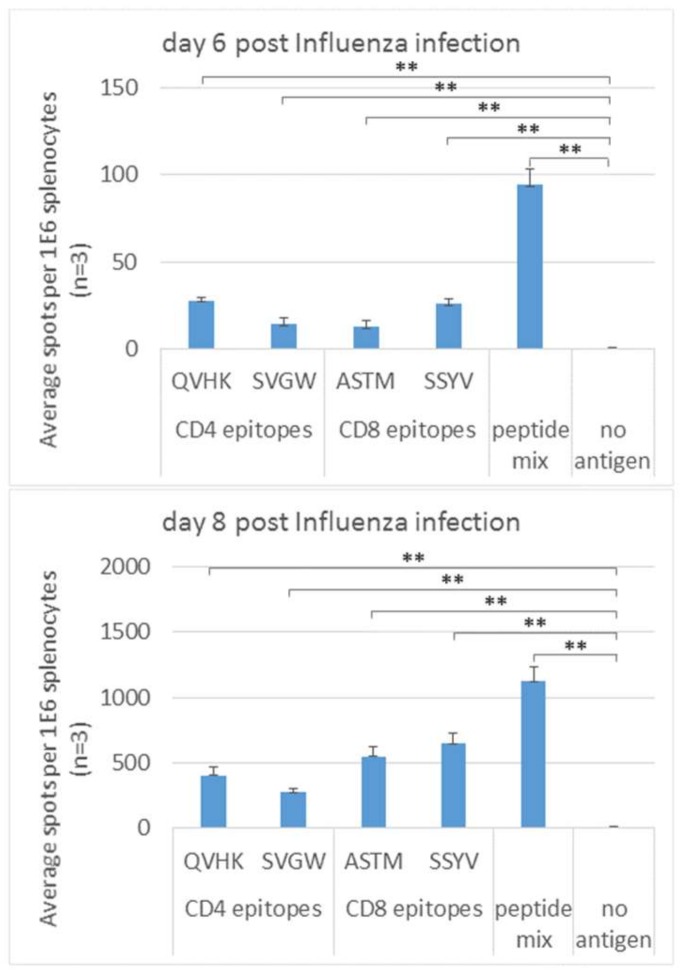
IFNγ ELISPOT response in the spleen following intranasal infection with Influenza strain A/Puerto Rico/8/1934 H1N1. Mice were infected with 20 pfu and groups of 3 spleens were collected in the indicated time points. Splenocytes were stimulated in situ in an ELISPOT assay with MHC peptide epitopes (1 µM total peptide concentration). Two immunodominant CD4 peptides epitopes (I-A^b^-restricted, NP311-325 QVYSLIRPNENPAHK, designated QVHK; NA161-175 SVAWSASACHDGMGW, designated SVGW), two CD8 peptide epitopes (D^b^-restricted, NP366-374 ASNENMETM, designated ASTM; PA224-233 SSLENFRAYV, designated SSYV) and a mixture of the four were used for stimulation. Data are representative of three independently performed experiments. ** *p* < 0.01.

The splenocytes response to a viral pathogen was examined in mice infected with the mouse-adapted Influenza Virus strain PR8. Mice splenocytes were harvested on day 4, 6 and 8 post infection. In this experimental system, the potential of immunodominant MHC-binding peptide antigens for stimulation in the ELISPOT assay, was examined. This approach potentially increases the specificity of the detected response and may exhibit lower background responses. The peptides used in the experiments were previously described as CD4 (I-A^b^-restricted) [19] and CD8 (D^b^-restricted) [20] T cell epitopes, encoded by the PR8 strain of Influenza. No response was observed on day 4 post infection. On day 6 post infection 10–30 IFNγ-producing cells per 10^6^ splenocytes for individual peptides were observed (Figure 3), while no spontaneous IFNγ secretion was detected (no antigen). No response to the stimulating peptides was observed at earlier time-points or in splenocytes of naive mice (three individual naïve mice, not shown). Using a mixture of the four stimulating peptides resulted in an additive response, allowing a higher signal amplitude. On day 8 post infection, more than 10 fold higher response of 250–650 IFNγ-producing cells per 10^6^ splenocytes was observed. The background response in the absence of peptide stimulation (no antigen) was less than 10 spots per 10^6^ splenocytes.

### 3.4. Early Response to FT is Detected in Peripheral Blood Cells

**Figure 4:**
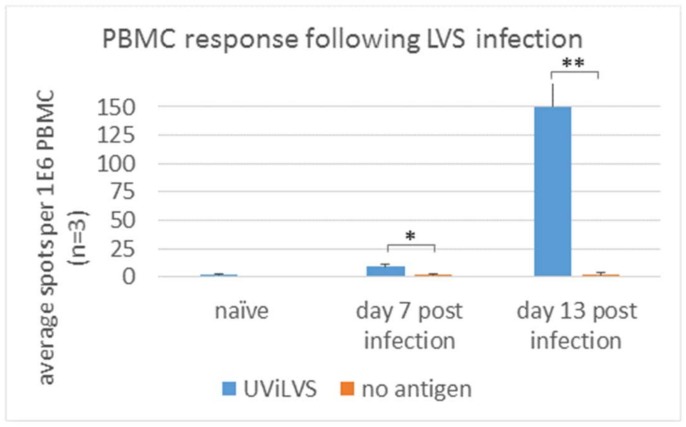
ELISPOT response in the blood following *F. tularensis* infection. Groups of three mice were infected intranasal with *F. tularensis* LVS (100 cfu) and bled 7 and 13 days post infection. PBMC from naïve and infected mice were stimulated with UViLVS as describe or left untreated. Data are representative of two independently performed experiments. * *p* < 0.05, ** *p* < 0.01.

The data indicated that an early immune response in the spleen could be detected upon infection with each of the four pathogens. Next, the possibility to detect the response in PBMC was determined following FT LVS infection (Figure 4). On day 7 post infection with 100 cfu LVS, we detected 5–10 IFNγ-producing cells per 10^6^ cells upon antigen stimulation, while no IFNγ-producing cells were observed in the non-stimulated cells. No response to the stimulating antigen was observed in PBMC from naïve, non-infected mice when stimulated with UViLVS (Figure 4). On day 13 post infection, the response was approximately 150 IFNγ-producing cells per 10^6^ cells, with background response from non-stimulated cells (spontaneous IFNγ secretion) below 5 spots. These data demonstrate that in the FT mouse model of infection, ELISPOT based on detection of IFNγ-producing cells may be carried out with blood samples.

### 3.5. Early Response in the Blood of NHP Inoculated with BA Spores

**Figure 5:**
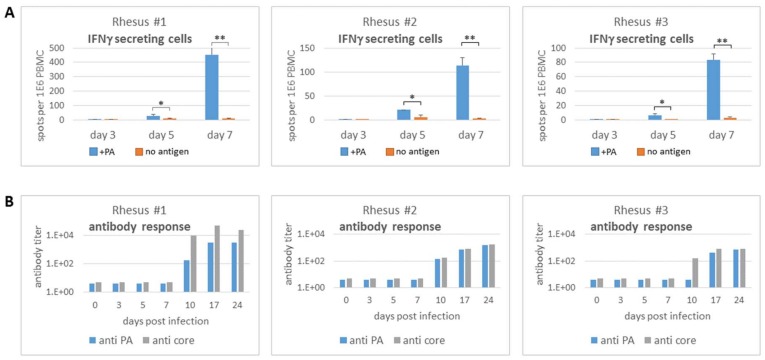
Cellular and humoral response in non-human primates following *B. anthracis* infection. Three individual animals were infected subcutaneously with 5 × 10^8^ spores of Sterne *ΔhtrA lefMUT Δcya* strain. The animals were bled in the indicated time points and their immune response was evaluated by ELISPOT (**A**) and ELISA (**B**) as described in the text. * *p* < 0.05, ** *p* < 0.01.

The data demonstrated the potential of ELISPOT assay for the detection of infection of various pathogens in the murine model. The assay was next applied in an NHP model, which recapitulates better the infections in humans. We recently described the development of a live, attenuated *B. anthracis* strain of Sterne subsp. It is mutated in the genes for *htrA*, *lef* and *cya* (Sterne *Δhtr Alef^MUT^ Δcya*), encoding for the stress response protein HtrA and LF and EF toxins, respectively [12]. Three individual rhesus macaques were inoculated sub-cutaneously with 10^9^ BA spores and blood samples were collected on day 3, 5 and 7 postinoculation. PBMC response to the protective antigen (PA) protein of *BA* was analyzed by ELISPOT and ELISA (Figure 5). No immune response was observed on day 3 post infection, while on day 5 post infection a significant ELISPOT response was observed for all three NHPs. Two days later, the ELISPOT response was 10–20 times stronger while the background response of spontaneous IFNγ secretion was not increased (10 spots per 10^6^ cells). The use of core antigen for antigen stimulation was examined, yet in this case, a high background response of naïve cells was observed (not shown), and therefore was considered not appropriate for specific diagnostic detection of infection. PA-specific antibodies and antibodies against bacterial core antigens were first observed 10 days after exposure to the bacteria (2 of 3 animals, Figure 5B). It is therefore concluded that the cellular immune response is observed considerably earlier than the antibody response, and can provide timely detection of pathogen infection.

### 3.6. Gene Expression Signature Can Serve for Detection of Early Immune Response

**Figure 6:**
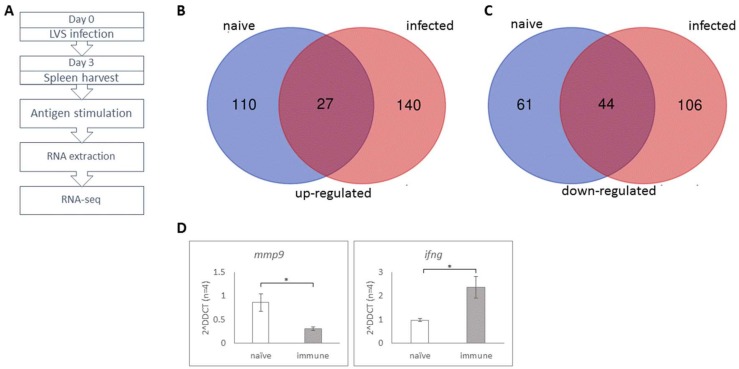
RNA-seq analysis. (**A**) Experimental flow chart: Splenocytes from five naive mice and five LVS-infected mice were stimulated in situ with UViLVS for 3 h. Total RNA was extracted and mRNA was sequenced. The number of genes that were significantly up-regulated (**B**) or down-regulated (**C**) in infected and naïve mice in response to antigen stimulation, as described in the text. (**D**) Real-time PCR validation of *mmp9* and *ifng* genes in splenocytes from LVS-infected versus naïve mice. The expression level was normalized to the *hprt* gene. * *p* < 0.05.

The ELISPOT assay requires at least 24 h for completion. In order to shorten the assay time and potentially increase its specificity, gene expression signature of antigen-stimulated splenocytes from FT LVS infected mice was examined. Mice were infected with 10^2^ cfu of FT LVS and their spleens harvested 3 days later. Splenocytes from infected and naïve mice were stimulated for 3 h with UViLVS or left unstimulated. Total RNA was extracted from the cells and examined by RNA-seq allowing a detailed mapping of transcripts levels in response to antigen stimulation (UViLVS) following infection with FT LVS (Figure 6A).

The expression pattern of 13,928 murine transcripts was quantitatively assessed (Table S1). Interestingly, in the non-stimulated groups, transcription of 786 genes was elevated by more than two-fold in response to FT LVS infection, reflecting the effect of the disease on splenocytes at that time point. Next, we compared the gene expression ratios of antigen-stimulated vs. non-stimulated samples, focusing on genes that were significantly changed (*p* value < 0.05) by at least 2 fold. Overall, in splenocytes from infected mice, 317 genes fit this criteria (Table S2). Among them 167 genes were elevated by the antigen stimulation and 150 were down-regulated by the stimulation. In splenocytes from naïve mice, 242 genes were significantly changed by more than 2 fold, among them the expression of 137 genes was elevated following antigen stimulation while the expression of 105 genes was decreased (Table S3).

Comparing between the genes that were up-regulated by antigen stimulation in the infected group (167 genes) to the genes that revealed the same extent of up-regulation in the naïve group (137 genes) (Figure 6B), identified 140 genes that were specifically and significantly elevated in the infected group. GO-term enrichment analysis for those 140 genes that were elevated only in the antigen stimulated infected group, was carried out. The group of up-regulated mRNAs was significantly enriched with genes related to inflammatory response, including cytokine mediated signaling pathway and positive regulation of cell proliferation. Specifically, up-regulation of several cytokine genes were identified, including IFNγ (9.3 fold increase) IL23 (7.6 fold), GM-CSF (4.6 fold) and IL-6 (2.6 fold), and chemokines such as CCL2 (2.9 fold), CCL7 (3.8 fold), CCL11 (15.0 fold) and CXCL5 (4.3 fold). While these proteins were previously found to be produced by activated T cells, the current experimental setup cannot associate the gene expression to a particular cell population.

Comparison between genes that were down-regulated by antigen stimulation in the infected group (150 genes) to the genes that showed the same extent of down-regulation in the naïve group (105 genes) (Figure 6C) identified 106 genes whose expression was specifically reduced in the infected group. Among them were the genes encoding for CEACAM1, NLPR12 and NLRC3, all of which were previously documented as negative regulators of T cell response [21,22,23,24].

Finally, RT-PCR was employed to validate the RNA-seq measurements, in biological replicate experiments from mice infected with FT LVS as described above. Real-time PCR analysis was performed with mRNAs from 4 individual mice for each group (antigen-stimulated cells from naïve and infected mice, and non-stimulated cells from naïve and infected mice). The expression of two genes distinguished by the RNA-seq analysis were inspected by RT-PCR: the gene *mmp9* whose transcription was significantly reduced by antigen stimulation in the infected group and the *ifng* gene whose transcription was strongly up-regulated by the stimulation following FT infection (Figure 6D). The independent measurements were in strong agreement with the RNA-seq data.

To summarize, the RNA-seq data provides a list of 140 genes that are specifically elevated and 105 genes that are down-regulated only in antigen-stimulated immune-primed mice. These genes can potentially serve as markers for a genetic-based immunoassay, in which monitoring the expression of chosen genes throughout antigenic T cell induction would indicate a specific T cell response.

## 4. Discussion

Early pathogen diagnosis is essential, and is of special importance in the context of emerging diseases and biothreat agents. Since direct isolation of the pathogen or its material is applicable only at a limited time-frame and sampling site, immunoassays for the detection of the immune response induced by a pathogen is required. Our current work is aimed at analyzing the potential of cellular immunity-based assays for the early detection of pathogen infection.

Pathogen infection leads to the development of acquired immunity, manifested by, among other effects, antibody secretion by B cells and cytokine secretion by T cells. Although immunoassays are limited in differentiating between memory response and recently acquired immunity, biothreat agents and emerging diseases mainly have a primary exposure nature. We looked for the time of immune induction in PBMCs following *Bacillus anthracis* infection by ELISA for serum antibodies and cellular immunity by ELISPOT assay for IFNγ secretion (Figure 5). While IFNγ secreting cells were spotted already five days following infection, serum antibodies were detected only 10 days post infection. We observed a similar trend when analyzing the immune response developed following infection of mice with a sub-lethal dose of *F. tularensis* strain LVS (not shown). This may be explained by the fact that ELISPOT allows the detection of low frequency cells (~10^−5^), while serum antibodies are accumulated and ELISA measures total analyte concentration. In accordance with this, antibodies are detected in the serum only 1–2 weeks following antigen exposure, while high-affinity antibodies are observed weeks later [25]. Therefore, for early detection, cellular immunity-based immunoassays are superior to serum antibodies assays such as ELISA.

IFNγ is produced by CD4 and CD8 T cells, NK and NKT cells, macrophages and dendritic cells [26]. The ELISPOT set-up was designed to allow specific T cell stimulation, with a mixed population of splenocytes or PBMC that includes antigen-presenting cells. Indeed, when stimulating with specific peptide epitopes we were able to detect both CD4 and CD8 response in Influenza-infected mice (Figure 3). Our previous studies of FT in the murine model showed IFNγ expression by CD4, CD8 and NK cells in the respiratory system as early as two days post intranasal infection [17]. The ELISPOT data reported here, cannot specify the source for IFNγ, however, a significant number of IFNγ-producing cells was dependent on both animal infection and antigen stimulation. NK cells may account for spontaneous, antigen-independent IFNγ secretion that was observed during the peak of the disease (“no antigen” response Figure 1C, day 6 post infection), and wained afterwards (day 12 post infection). This observation is in discordance with the typical evolving kinetics of acquired immune response that is usually observed in antigen-stimulated cells. Furthermore, in a previous study we observed memory response exhibiting significant boost-mediated augmentation, following FT infection [27]. Altogether, the data support the assumption that the cells secreting IFNγ- detected in the assays described in this study are mostly T cells, with a limited response in the peak of the disease attributed to NK cells. Clinical immunodiagnosis is mostly performed by ELISA to identify serum antibodies. There are some limitations for ELISA in the context of emerging diseases. The assay is dependent on the availability of antigens that retain the 3D structure of the pathogen epitopes. Conversely, monitoring T cell response in situ could be performed with linear peptides. Moreover, unique protein sequences could be synthesized for specific stimulating, to avoid potential cross-reaction with other known pathogens. Similar methodology was demonstrated in the case of DENV- and ZIKV-infected individuals, where ELISPOT assay was described to differentiate between infections with the two viruses with high sensitivity and specificity [28]. Another example for selecting appropriate stimulating antigens for specific pathogen identification is the “T-Spot. TB” commercial kit for identification of tuberculosis. The stimulating antigens in the assay are ESAT-6 and CFP-10 proteins, to allow the specific identification of tuberculosis, distinguishable from BCG and most other non-tuberculous mycobacteria [9]. The choice of the appropriate antigens is essential for a specific assay with minimal background, to eliminate potential false positive signals from naïve individuals. As observed here and also by others, stimulation with subunit antigens (proteins, peptides etc.), yields minimal background compared to neutralized whole-pathogen. A study performed in the years 2002 to 2004 showed a limited use of ELISPOT for the diagnosis of Lyme disease due to high IFNγ secretion by cells from non-infected individuals. However, the stimulating antigen in the assay was an outer surface protein-enriched fraction from *Borrelia burgdorferi* which may cross react with other bacterial antigens [29]. Indeed, in a later study, higher specificity was observed [30], partially owing to the use of recombinant *Borrelia burgdorferi* proteins as antigens. As seen in the current study, the use of MHC-binding peptide epitopes enabled us to monitor PR8 infection already 6 days post infection (Figure 3). The use of a mix of epitopes was, as expected, additive to the use of each epitope individually. In the case of emerging disease, pathogen-specific MHC epitopes can be identified bioinformatically and serve as stimulating antigens in a T-cell-based immunoassay. That concept was demonstrated for Zika virus where candidate targets for Zika-specific immunity were identified through comparison to known protein sequences of other Flaviruses [31].

Our current study elaborates on ELISPOT assays for identification of IFNγ-secreting cells in response to antigen in situ. IFNγ expression correlates with a high hierarchy and quality of T cell effector function among cytokine-expressing cells and with protective immunity [2]. In addition to IFNγ, we evaluated the secretion of two additional cytokines involved in the immune response, IL-2 and TNFα (data not shown). However, these were found to be unsuitable for our assays. In the case of IL-2, we observed antigen-independent IL-2 release in infected mice at certain time points, irrespective of antigenic stimulation, masking the antigen-specific response. In the case of TNFα, we observed cytokine secretion in splenocytes from non-infected mice, in response to stimulation with antigens in the form neutralized whole pathogens (UViLVS and *B. anthracis* core). TNFα secretion is induced by TLR triggering of different cell types, through pathogen-associated molecular patterns (PAMP) recognition by pathogen-associated receptors (PRR). Choosing the appropriate antigen for stimulation is essential to minimize such background. Additionally, methods other than ELISPOT, such as intracellular staining (ICS) for flow cytometry can correlate cytokine production to a specific cell type. Several reports have shown that expression of cytokines by polyfunctional cells, identified by ICS, correlates with protection, such as in the case of TB [32] and *Leishmania* infection [33]. ELISPOT and flow cytometry data were found to correlate on non-specific stimulation of human PBMC [34]. However, ELISPOT is advantageous for its sensitivity and simplicity [6,35], and therefore is suitable for robust immunoassays.

The common procedure for ELISPOT assay requires at least 24 h for completion. Although the incubation time for T cell activation in situ may be shortened ([35] and our unpublished data), it lowers the number of detected spots and therefore diminishes the assay’s sensitivity. Methods for the detection of specifically activated cells are also based on other cell characteristics, such as physical shape, Ca2+ mobilization and gene expression [6,36,37]. Activation-induced markers (AIM) were used to identify antigen-specific T cells in a cytokine-independent assay [38,39,40], to detect vaccine and natural infection-induced CD4 T cell responses. Using a different approach, electrical impedance was shown to detect physical and structural shape changes upon T cell activation within 10–30 min [41,42]. However, this has been demonstrated under robust and coherent stimulation conditions, under a limited acquisition rate that is unsuitable for the detection of low-frequency events [43].

Our gene expression analysis showed antigen-dependent, specific response in immune primed cells. We identified 140 genes that are up-regulated and 106 down-regulated in antigen-stimulated cells from infected mice and not in antigen-stimulated naïve cells. Future experiments are required to validate our data with other pathogen infections and antigen types, in order to assess their potential as markers in a gene-based immunoassay. In such assays, RT-PCR can serve for monitoring the change of expression of certain genes, to correlate with specific T cell stimulation. Additionally, this approach could be supported by correlating gene expression to T cells, and monitoring the kinetics of gene expression during in situ antigen stimulation [44] could support the data validation.

Altogether, we show here that cell-based immunoassays are a suitable tool for the identification of pathogen infection. They can identify infection earlier than the conventional ELISA assay for serum antibodies detection, and gene-expression signature can potentially serve to shorten the assay.

## Supplementary Materials

The following are available at https://www.mdpi.com/2073-4409/8/9/952/s1. Table S1: Fold change and significance value (*p*-value) of transcripts from splenocytes of naïve or infected mice that were either stimulated by antigen or left unstimulated. Table S2: Transcripts that were significantly up-regulated or down-regulated (*p*-value < 0.05) by at least 2 fold in splenocytes from infected mice. Table S3: Transcripts that were significantly up-regulated or down-regulated (*p*-value < 0.05) by at least 2 fold in splenocytes from Naive mice.
